# Effect of opportunistic salpingectomy at hysterectomy on anti‐Müllerian hormone: A substudy of a randomized trial

**DOI:** 10.1111/aogs.70247

**Published:** 2026-05-19

**Authors:** Anna Darelius, Annika Idahl, Karin Sundfeldt, Annika Strandell

**Affiliations:** ^1^ Department of Obstetrics and Gynecology, Institute of Clinical Sciences, Sahlgrenska Academy University of Gothenburg Gothenburg Sweden; ^2^ Region Västra Götaland, Department of Obstetrics and Gynecology Sahlgrenska University Hospital Gothenburg Sweden; ^3^ Department of Clinical Sciences, Obstetrics and Gynecology Umeå University Umeå Sweden

**Keywords:** anti‐Müllerian hormone, epithelial ovarian cancer, hysterectomy, opportunistic salpingectomy, ovarian function

## Abstract

**Introduction:**

Opportunistic salpingectomy during benign hysterectomy with ovarian preservation has become a preventive approach to epithelial ovarian cancer, particularly high‐grade serous carcinoma, given evidence that it originates in the Fallopian tubes. However, the long‐term impact of the procedure on ovarian function remains unknown. This study assessed whether opportunistic salpingectomy at benign hysterectomy affects ovarian function compared with no salpingectomy 1 year after surgery.

**Material and Methods:**

This is a noninferiority substudy of the national register‐based randomized controlled trial HOPPSA (Hysterectomy and OPPortunistic SAlpingectomy), registered at clinicaltrials.gov (NCT03045965) on 8 February 2017. Women under 55 years planned for a benign hysterectomy at the Sahlgrenska University Hospital were randomized to hysterectomy with opportunistic salpingectomy or hysterectomy‐only. Blood samples were collected preoperatively and 1 year postoperatively and analyzed for anti‐Müllerian hormone (AMH) by Access2 immunoassay. The primary outcome was absolute change in AMH over time in the per‐protocol population. Secondary outcomes were the relative change in AMH and level of AMH 1 year after surgery. The difference between groups was estimated by analysis of covariance, adjusted for baseline AMH, age groups, operative route, and time from surgery to second sampling. Multiple imputation was applied on missing samples. The noninferiority margin was set to 0.125 μg/L.

**Results:**

Between 16 August 2018 and 6 September 2023, 89 women were randomized to hysterectomy with opportunistic salpingectomy (*n* = 46) or hysterectomy‐only (*n* = 43). After exclusions due to prior ovarian surgery or not receiving the allocated surgery, 37 women were analyzed in each group. Mean age was 45.1 and 46.3 years in respective groups and mean body mass index was 29.5 and 27.2 kg/m^2^. Baseline characteristics did not differ between the groups. Both groups had reduced AMH levels 1 year postoperatively. The adjusted mean difference in the reduction was −0.03 μg/L (95% CI −0.23 to 0.18), with 0.18 exceeding the predefined noninferiority limit.

**Conclusions:**

Noninferiority was not demonstrated for opportunistic salpingectomy at hysterectomy compared with hysterectomy‐only regarding ovarian function assessed by AMH. Absolute reduction in AMH levels appeared to be similar in both groups, with an unexpectedly large variability in AMH reduction suggesting a more pronounced decline in some individuals.

## Graphical abstract

Noninferiority of opportunistic salpingectomy during benign hysterectomy compared with hysterectomy‐only concerning ovarian function measured by anti‐Müllerian hormone 1 year after surgery could not be declared. Studies with clinical outcomes related to ovarian function are still needed.

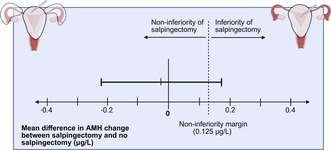

**Abbreviations**
AMH: anti‐Müllerian hormone
CI: confidence interval
EOC: epithelial ovarian cancer
GynOp: The Swedish National Quality Register of Gynecological Surgery
HOPPSA: Hysterectomy and OPPortunistic Salpingectomy
ITT: intention to treat
OS: opportunistic salpingectomy
SD: standard deviation

**Key message**

Noninferiority of opportunistic salpingectomy during benign hysterectomy compared with hysterectomy‐only concerning ovarian function measured by anti‐Müllerian hormone before and 1 year after surgery could not be declared. Studies with clinical outcomes related to ovarian function are still needed.

## 1 INTRODUCTION

Opportunistic salpingectomy (OS) during benign hysterectomy with ovarian preservation has emerged as a preventive intervention for epithelial ovarian cancer (EOC), particularly high‐grade serous carcinoma, which originates from the Fallopian tube epithelium.^1^, ^2^ Large observational studies have shown a significant reduction (42%–65%) in EOC risk after nonopportunistic bilateral salpingectomy compared with no surgery.^3^, ^4^ OS at the time of benign hysterectomy has been increasingly performed in Sweden^5^ and elsewhere,^6^, ^7^, ^8^ although the risks and complications have not been studied sufficiently.^9^, ^10^ Moreover, the actual EOC risk reduction after OS at hysterectomy is not known.

Although the preventive benefits of OS are promising, concerns have been raised regarding the potential impact of OS on ovarian function and the risk of lower age at menopause. OS involves surgery close to the ovaries and may damage ovarian blood vessels, increasing the risk of impaired ovarian function.^11^, ^12^ Each year of earlier menopause is associated with incremental increases in long‐term health risks, including cardiovascular disease, osteoporosis, cognitive decline, and an increased risk of all‐cause mortality.^13^, ^14^, ^15^, ^16^ Up to date no studies exist on the effect of OS on timing of menopause. Short‐term studies on hysterectomy with OS using surrogate measures for ovarian function have generally shown no significant impact on ovarian function.^10^, ^17^ On the other hand, one Swedish study shows a higher risk of menopause symptoms 1 year after hysterectomy with opportunistic salpingectomy compared with hysterectomy only.^5^ However, the long‐term effects on ovarian function remain unexplored.

There is currently no reliable biomarker to predict the timing of menopause. Anti‐Müllerian hormone (AMH) strongly correlates with the ovarian follicle pool and ovarian function.^18^

Given that the long‐term effects of OS on ovarian function are not yet fully understood, and a temporary decrease in AMH has been observed within 3 months following hysterectomy,^19^ it is crucial with an extended follow‐up. It is crucial to understanding the long‐term effects of OS to ensure that the potential benefits of its application as a preventive procedure during benign hysterectomy for ovarian cancer risk reduction do not incur detrimental effects on ovarian function or precipitate earlier menopause. This is particularly important before recommending the additional procedure to women at average risk of ovarian cancer planned for benign hysterectomy, given the well‐known health burdens associated with early menopause. The aim of this study was to assess whether OS at benign hysterectomy affects ovarian function measured by AMH 1 year postoperatively.

## 2 MATERIAL AND METHODS

This is a single‐center noninferiority substudy of the national register‐based randomized controlled trial HOPPSA (Hysterectomy and OPPortunistic SAlpingectomy), registered at clinicaltrials.gov (NCT03045965) on 8 February 2017.

### 2.1 The HOPPSA trial

HOPPSA aimed to investigate the effect of OS at the time of benign hysterectomy on the risk of surgical complications, menopause symptoms, and risk of EOC.^20^ Women aged <55 years planned to undergo benign hysterectomy were screened for eligibility within the Swedish National Quality Register of Gynecological Surgery (GynOp). Eligible and consenting patients were randomized to hysterectomy with OS or hysterectomy‐only.

Randomization was performed in a 1:1 ratio using a dedicated randomization module within GynOp as close to the time of surgery as possible. Even distribution between randomization groups over time was ensured by applying variable block sizes. All operative routes (abdominal, laparoscopic, and vaginal) were included. GynOp contains data on each patient's health background, surgical indications, the extent of surgery, perioperative and postoperative complications, and patient satisfaction. Exclusion criteria were previous or planned bilateral oophorectomy or salpingectomy, or not understanding oral or written study information.

### 2.2 The AMH substudy

Women consenting to HOPPSA at Sahlgrenska University Hospital were invited to participate in this substudy. Serum‐AMH levels were obtained by venipuncture at baseline and 1 year postoperatively. Separate written informed consent was obtained during the preoperative visit. Allocation to the intervention (hysterectomy with OS) or reference (hysterectomy‐only) group was achieved by the HOPPSA randomization. All patients received standard preoperative and postoperative care according to hospital protocol. Consenting patients had a blood sample drawn at the preoperative visit or on the day of surgery. One year after the first blood sample, the participants were contacted by phone and received an appointment for a second blood sample. Additional phone calls were made to nonresponders at the time of the second blood sampling and at trial completion. Blood samples were centrifuged at 2300 *g* for 10 min at room temperature within 30 min of collection. The separated serum was frozen and stored in a biobank at −70°C for the duration of the trial. After trial completion, all blood samples were analyzed at the Department of Clinical Chemistry, Region Västra Götaland, Sahlgrenska University Hospital, Gothenburg. The serum levels of AMH were measured using the Access2 immunoassay system (Access AMH), a chemiluminescence immunoassay with paramagnetic particles (Beckman Coulter, Brea, CA, USA). The limit of quantitation was 0.02 μg/L. The inter‐assay coefficients of variation for Access AMH were ≤6.6% at a serum level of 0.96 μg/L and ≤5% at a serum level of 4.5 μg/L. The blood samples were analyzed on two different occasions because the target number of participants was not reached after the first inclusion process, and an extended recruitment period was needed. Each participant's two samples were analyzed on the same occasion, and the same assay was used for all participants. Demographic variables were extracted from the GynOp register, including age, body mass index (BMI), parity, use of hormonal contraceptives or any medication with estrogen for menopause symptoms, smoking habits, intended and actual surgical route, indication for hysterectomy, physical status according to the American Society of Anesthesiologists (ASA) classification, gynecological surgery or diagnoses prior to the surgery, and other gynecological surgical interventions during the follow‐up. The duration of surgery, perioperative complications, and blood loss were treated as descriptive covariates and were also retrieved from GynOp.

### 2.3 General statistics

The noninferiority design utilizes the per protocol population for the primary analysis. The noninferiority margin for the difference in the change from preoperative to postoperative serum‐AMH levels (expected reduction) between groups was defined as 0.125 μg/L, based on earlier studies on age‐related levels and decline. Morelli et al. reported a preoperative AMH of 0.5 μg/L in women with a mean age of approximately 46 years.^21^ Trabuco et al. reported a median relative decline of 40.7% of AMH 1 year after hysterectomy.^22^ Based on these previous studies, mean baseline AMH was estimated to be 0.5 μg/L decreasing to 0.25 μg/L 1 year postoperatively in the hysterectomy only group, corresponding to an absolute change of −0.25 μg/L. The noninferiority margin for the difference between groups was set to 0.125 μg/L, representing 50% of the anticipated decline due to hysterectomy and aging. Non‐inferiority could be declared if the upper limit of the two‐sided 95% confidence interval (CI) for the absolute difference in the change between the two groups did not exceed the predefined noninferiority margin.

### 2.4 Sample size

Assuming an SD of 0.1 μg/L for the change in AMH (as reported by Morelli et al.)^21^ and estimating the reduction in AMH levels being up to 0.05 μg/L greater in the intervention group, 29 patients were needed per randomization group to achieve a power of 80% (*β* = 20%). Estimating a 20% loss to follow‐up (a second blood sample not taken), at least 74 patients were required to be enrolled.

### 2.5 Populations

The intention‐to‐treat (ITT) population was defined as all randomized subjects with a first blood sample taken. The per protocol population originating from the ITT population included all subjects who underwent the allocated surgical intervention. The as‐treated population was not defined by the randomized groups, but according to what surgical intervention was performed. Thus, in the as‐treated population, the OS group includes all women with no Fallopian tube left in situ and the hysterectomy‐only group includes women with at least one tube remaining. Women who underwent any ovarian surgery during the hysterectomy or follow‐up were excluded from the per‐protocol and as‐treated populations.

### 2.6 Statistical analyses

The primary outcome was the absolute change in AMH over the 1‐year period. The comparison between groups was expressed as the difference between hysterectomy with OS and hysterectomy‐only. It was estimated with a 95% CI using an analysis of covariance (ANCOVA) model adjusted for baseline AMH, age groups (<50 or ≥50 years), intended surgical route, and time from surgery to the second sampling of AMH. Missing data on the second blood sample was replaced with multiple imputation using fully conditional specification with 50 datasets, an initial seed of 132 557, and based on the following variables: group (intervention/reference), age at surgery, surgical route (abdominal/laparoscopic/vaginal), operative time, baseline AMH, perioperative blood loss, number of days from surgery to second blood sampling, and AMH levels at second sampling. An additional unadjusted analysis was performed in the per‐protocol population. A sensitivity analysis of complete cases and complementary analyses of the ITT and as‐treated populations were performed by applying the adjusted model described above. For the complementary analyses, multiple imputation for missing data on the second sample was used.

Secondary outcomes were the relative change in AMH and level of AMH 1 year after surgery. The outcomes were tested using the ANCOVA model adjusted for baseline AMH, age groups, intended operative route, and time from surgery to the second sampling of AMH. Multiple imputation was applied. Exploratory analyses involved age stratification (<45, 45–49, and ≥50 years) within the per‐protocol population using the same adjusted model as in the primary analysis, with complete cases and multiple imputation for missing values in the second blood sample. Prior to analyses, a statistical analysis plan was published on www.clinicaltrials.gov. SAS® version 9.4 TS Level 1M6 (Cary, NC, USA) was used.

## 3 RESULTS

**FIGURE 1:**
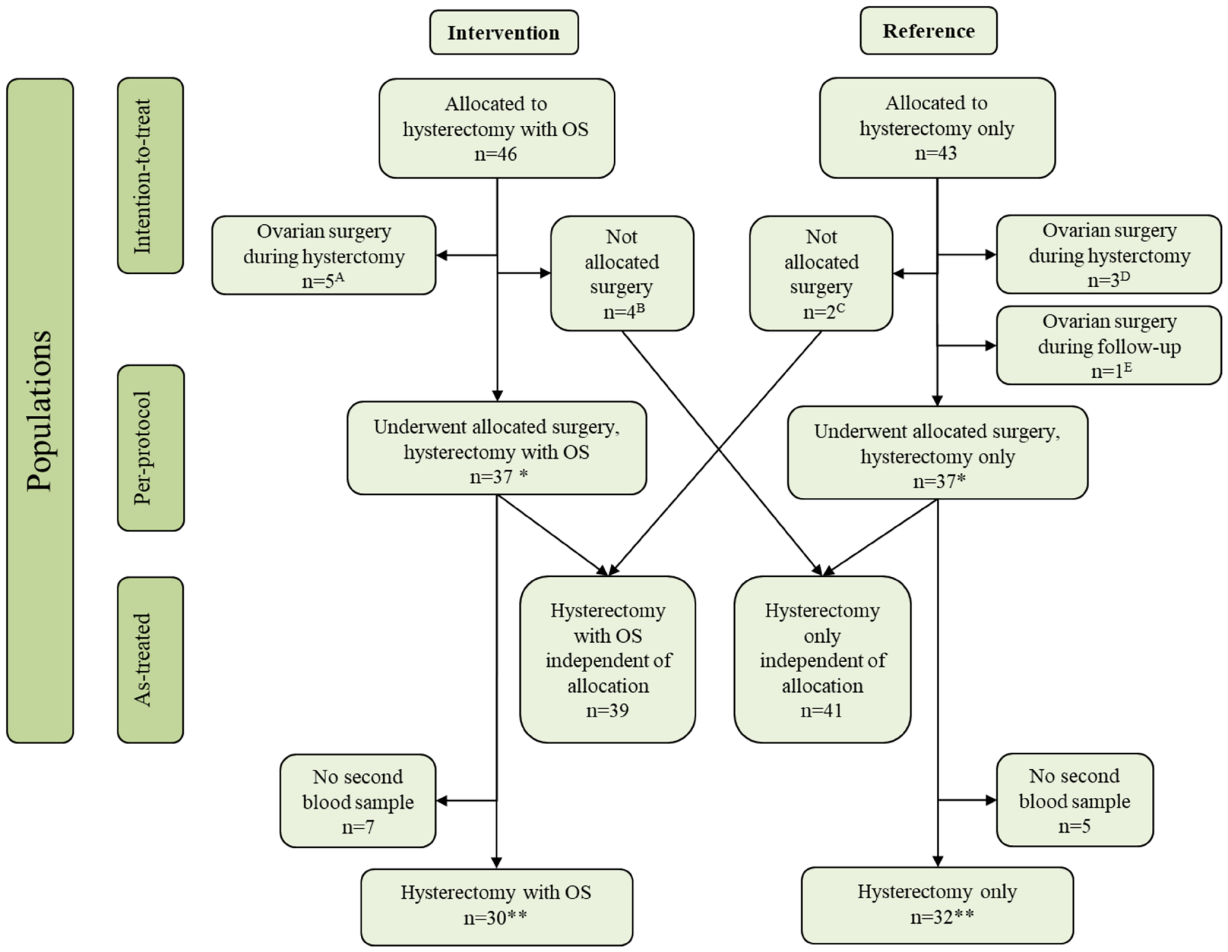
Flow diagram of patient inclusion. OS, opportunistic salpingectomy. ^A^Fenestration or enucleation of ovarian cysts (*n* = 4) and bilateral salpingo‐oophorectomy due to moderate to severe endometriosis (*n* = 1). ^B^OS was not performed due to adherence status based on severe to moderate endometriosis (*n* = 2) or no specified reason (*n* = 2). ^C^OS was performed due to unspecified pathology (*n* = 2). ^D^Enucleation of ovarian cysts (*n* = 2) and unilateral salpingo‐oophorectomy due to endometriosis (*n* = 1). ^E^Fenestration of an ovarian cyst. *Included in primary analysis. **Included in sensitivity analysis.

The randomized HOPPSA cohort at Sahlgrenska University Hospital included 254 women during the substudy period. Between 16 August 2018 and 6 September 2023, a total of 89 women were recruited from the cohort and randomized to hysterectomy with OS (*n* = 46) or hysterectomy‐only (*n* = 43), forming the ITT population (Figure 1). In the OS group, five women from the ITT population were excluded due to ovarian surgery during hysterectomy and four did not receive the allocated surgery. In the hysterectomy‐only group, four women were excluded from the ITT population due to ovarian surgery during either hysterectomy or follow‐up, and two did not receive the allocated surgery; the reasons are presented in Figure 1. Prior to hysterectomy, two women in the OS group had a previous unilateral salpingectomy and one woman in the hysterectomy‐only group had a previous unilateral oophorectomy; all three individuals were included in the per‐protocol population. Therefore, 74 women constituted the per‐protocol population, 37 in each group.

Women in the OS group were slightly younger (mean age 45.1 vs. 46.3 years), had a higher BMI (mean 29.5 kg/m^2^ vs. 27.2 kg/m^2^) and were more commonly current smokers (20% vs. 14%) than the women in the hysterectomy‐only group (Table 1). None of these differences were significant. The most frequent indication for hysterectomy was uterine fibroids with bleeding disorder, pressure or pain, or no information on symptoms (84.9%, *n* = 62). Other indications were menorrhagia or metrorrhagia (9.6%, *n* = 7) and dysmenorrhea (5.5%, *n* = 4). Most women underwent laparoscopic hysterectomy (86.5%, *n* = 64), and 10 (13.5%) underwent abdominal hysterectomy. The OS group experienced an average of 11 min longer operative time (95% CI −9.2 to 32.1), and 20 mL larger perioperative blood loss (95% CI −39.8 to 79.8) compared with the hysterectomy‐only group. A second blood sample was missing for 19% of patients in the OS group and 14% of patients in the hysterectomy‐only group. A drop‐out analysis did not reveal any differences in the baseline characteristics or perioperative covariates between patients with and without a second blood sample.

**TABLE 1:** Baseline characteristics and per‐operative covariates in the per‐protocol population.

Variable	Intervention	Control	Missing
	Hysterectomy with OS	Hysterectomy only	
	*n* = 37	*n* = 37	I/C
Age (years) mean (SD)	45.1 (5.3)	46.3 (4.7)	0/0
Median (Q1; Q3)	46.0 (41.5; 49.5)	47.0 (43.0; 50.0)	
Body mass index (kg/m^2^)	29.5 (5.2)	27.2 (4.7)	3 (8.1%)/1 (2.7%)
Smoking status			
1–5/day	5 (14.3%)	2 (5.6%)	2 (5.4%)/1 (2.7%)
>5/day	2 (5.7%)	3 (8.3%)	
No, have quit	10 (28.6%)	9 (25.0%)	
No, have never smoked	18 (51.4%)	22 (61.1%)	
Parity			
0	6 (17.1%)	5 (14.3%)	2 (5.4%)/2 (5.4%)
1	4 (11.4%)	8 (22.9%)	
2	17 (48.6%)	15 (42.9%)	
≥3	8 (22.9%)	7 (20.0%)	
Preoperative ASA^a^ classification			
ASA 1	16 (43.2%)	24 (64.9%)	0/0
ASA 2	20 (54.1%)	13 (35.1%)	
ASA 3	1 (2.7%)	0	
Prior gynecological disease			
Salpingitis	1 (3.0%)	1 (3.1%)	4 (10.8%)/5 (13.5%)
Endometriosis	1 (3.0%)	1 (3.1%)	4 (10.8%)/5 (13.5%)
Ovarian cysts	6 (18.2%)	3 (9.3%)	4 (10.8%)/5 (13.5%)
Fibroids	28 (82.4%)	25 (71.4%)	3 (8.1%)/2 (5.4%)
Prior abdominopelvic surgery			
Caesarean section	9 (26.5%)	9 (25.7%)	3 (8.1%)/2 (5.4%)
Sterilization	4 (11.8%)	5 (14.7%)	3 (8.1%)/3 (8.1%)
Ectopic pregnancy	3 (8.8%)	0 (0%)	3 (8.1%)/3 (8.1%)
Adnexal surgery	2 (5.9%)	2 (5.9%)	3 (8.1%)/3 (8.1%)
Myomectomy	4 (11.8%)	9 (25.7%)	3 (8.1%)/2 (5.4%)
Use of any contraceptives			
Yes	28 (84.8%)	28 (93.3%)	4 (10.8%)/7 (18.9%)
No	5 (15.2%)	2 (6.7%)	
Duration of hormonal contraceptives (years)	9.3 (9.0)	11.9 (11.4)	10 (27.0%)/12 (32.4%)
Menopausal symptoms prior surgery			
Yes	4 (14.8%)	8 (25.8%)	2 (5.4%) /1 (2.7%)
No	15 (55.6%)	15 (48.4%)	
Do not know	8 (29.6%)	8 (25.8%)	
Did not receive the question^b^			8 (5.4%)/5 (13.5%)
Menopausal estrogen treatment pre‐op			
Yes	1 (3.7%)	3 (10.0%)	2 (5.4%)/2 (5.4%)
No	26 (96.3%)	27 (90.0%)	
Did not receive the question^b^			8 (21.6%)/5 (13.5%)
Menopausal estrogen treatment 1‐year postoperative			
Yes	2 (6.5%)	4 (12.5%)	5 (13.5%)/5 (13.5%)
No	29 (93.5%)	28 (87.5%)	
Did not receive the question^b^			0/1 (2.7%)
Indication for hysterectomy			
Uterine fibroids	29 (80.6%)	33 (89.2%)	1 (2.7%)/0
Meno‐metrorrhagia	4 (11.1%)	3 (8.1%)	
Dysmenorrhea	3 (8.3%)	1 (2.7%)	
Primary incision			
Abdominal	3 (8.1%)	6 (16.2%)	0/0
Laparoscopic	34 (91.9%)	31 (83.8%)	
Conversion to abdominal surgery			
Yes	1 (2.9%)	0	0/0
Duration of surgery (min)	141.3 (52.7)	129.8 (34.7)	0/0
Per‐operative bleeding (ml)	103.9 (154.5)	83.9 (93.4)	0/0
Per‐operative complication	0	1 (2.7)	0/0
Time from surgery to second blood sampling (days)	401 (149.7)	383 (67.8)	7 (18.9%)/5 (13.5%)

*Note*: Data are reported as mean (SD) or as *n* (% of valid). Missing values are expressed as *n* (% of total) in the respective groups.

Abbreviations: C, control; I, intervention; OS, opportunistic salpingectomy.

^a^ Physical status classification system according to American Society of Anesthesiologists.

^b^ Women aged <40 do not receive question about menopausal symptoms.

The primary outcome, absolute change in AMH, was analyzed in the per‐protocol population from imputed data. The preoperative mean AMH levels did not differ significantly between the OS and hysterectomy‐only groups (1.01 μg/L vs. 0.88 μg/L). Both groups had reduced AMH levels 1 year postoperatively (secondary outcome) with an adjusted mean postoperative AMH of 0.70 μg/L vs. 0.67 μg/L in the OS and hysterectomy‐only groups, respectively.

**FIGURE 2:**
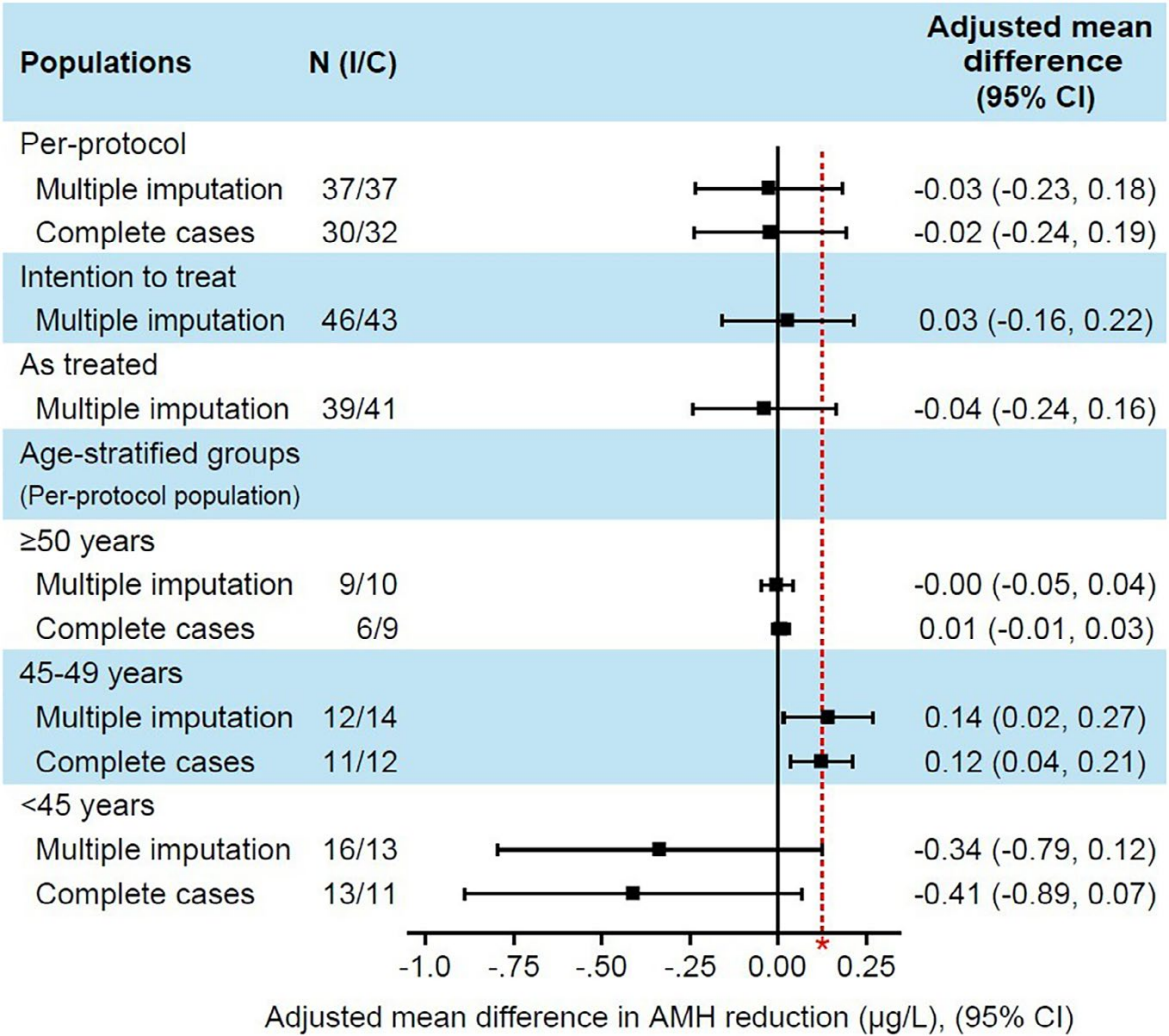
Adjusted mean difference in AMH reduction over time per population and in age‐stratified groups. *Noninferiority margin of 0.125 μg/L regarding the upper limit of 95%CI I, intervention (hysterectomy with salpingectomy) R, reference (hysterectomy only).

The corresponding absolute changes were −0.25 μg/L vs. −0.28 μg/L (Table S1). The primary outcome, the adjusted mean difference in reduction, was −0.03 μg/L (95% CI −0.23 to 0.18) (Figure 2). The upper limit for the CI (0.18 μg/L) exceeded the predefined margin of 0.125 μg/L, stating that noninferiority cannot be confirmed. The unadjusted analysis had similar results. Absolute changes were −0.27 μg/L and −0.26 μg/L in the OS and hysterectomy‐only groups, and the mean difference in reduction was 0.01 μg/L (95% CI −0.28 to 0.30).

**FIGURE 3:**
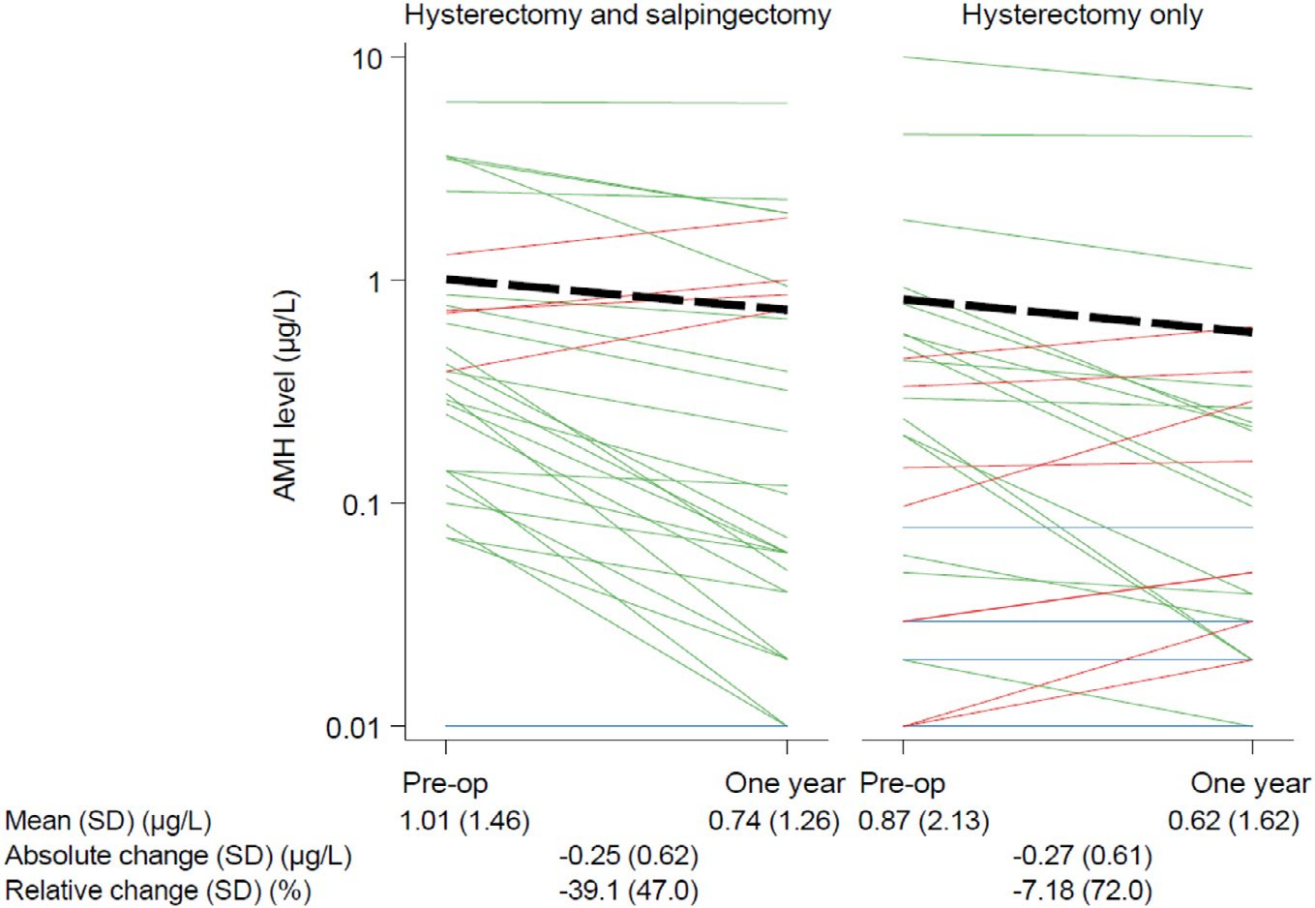
Change in anti‐Müllerian hormone (AMH) over time in the per‐protocol population (complete cases).

The relative change in AMH (a secondary outcome) was −14.3% (SD 47.0) vs. −9.75% (SD 72.0) in the OS and hysterectomy‐only groups (Table S1), with an adjusted mean difference of −4.55 percentage points (95% CI −78.9 to 69.8). The sensitivity analysis on complete cases (*n* = 62) produced similar results, with a mean absolute change in AMH of −0.25 μg/L (SD 0.62) in the OS group and −0.27 μg/L (SD 0.61) in the hysterectomy‐only group (Table S1 and Figure 3). The adjusted mean difference in the reduction was −0.02 μg/L (95% CI −0.24 to 0.19) (Figure 2). The relative change was −39.1% (SD 47.0) vs. −7.18% (SD 72.0) in the OS and hysterectomy‐only groups, respectively (Table S1). The mean adjusted difference was −31.9 percentage points (95% CI −63.9 to 0.12).

Complementary analyses on the ITT and as‐treated populations had similar results as in the per‐protocol population (Tables S2 and S3). The differences in the absolute change in AMH between groups are presented in Figure 2.

The exploratory age‐stratified analyses are presented in Figure 2, showing the adjusted mean differences in the absolute change in AMH between groups. Data for each randomization group are provided in Tables S4–S6.

## 4 DISCUSSION

Noninferiority of OS at hysterectomy compared with hysterectomy‐only regarding the decline in AMH at the 1‐year follow‐up could not be confirmed. Mean levels and absolute changes appeared to be similar in the two surgical groups, but large variability resulted in a wide CI, exceeding the pre‐defined non‐inferiority limit. The secondary outcome, the relative change in AMH, was somewhat larger in the OS group, which should be interpreted with great caution. A small absolute change in women with very low AMH levels yields a large relative change without clinical relevance. In the exploratory age‐stratified analyses, OS seemed to have less of an impact on the reduction of AMH in younger women. However, these findings are limited by the very small sample size within each subgroup. Recent studies comparing hysterectomy with and without OS show no short‐term effect on ovarian function based on AMH measurements.^23^, ^24^, ^25^, ^26^ Most studies suffer from small sample sizes, short follow‐up, and non‐randomized design, leaving the long‐term effect on the ovaries uncertain. A Dutch randomized controlled trial with a 6‐month follow‐up found no significant difference in the change in median AMH between hysterectomy with and without OS.^27^ Similarly, a retrospective cohort study by Morelli et al. found no significant difference in the change in AMH after hysterectomy with OS compared with hysterectomy‐only, though their study reported less variation in the changes in AMH (SD 0.1 vs. 0.6 in our study).^21^ To the best of our knowledge, no previous study has expressed a hypothesis of noninferiority and applied such a design. When assessing the relative change in AMH, our findings align with those of Song et al. and Van Lieshout et al., who reported a greater relative decrease in AMH in the OS group compared with the hysterectomy‐only group, with declines of 13 vs. 11 percentage points at 3 months^23^ and 4 vs. 0 percentage points at 6 months.^27^

The effects on ovarian function after OS have also been evaluated using non‐hormonal assessment methods. A Canadian study found no increased risk of physician visits related to menopause symptoms or prescription of menopausal hormone treatment among women who underwent OS at the time of hysterectomy.^28^ On the other hand, a Swedish cohort study suggested a 33% increased risk of experiencing menopause symptoms 1 year after hysterectomy with OS compared with hysterectomy‐only.^5^

High‐quality evidence on risks of OS is required before recommending it as a preventive strategy for EOC in women at average risk of ovarian cancer planned for benign hysterectomy. If risk is associated with OS, women must be informed to enable shared decision‐making regarding the removal of healthy organs. Although this study did not demonstrate non‐inferiority of OS during hysterectomy with respect to ovarian function at 1 year postoperatively, no detrimental effect was apparent, as the absolute reduction in AMH was comparable between the groups. Given the substantial inter‐individual variability in AMH reduction, indicating that some individuals may experience a more pronounced decline, larger studies examining clinical measures of ovarian function are needed before recommending the procedure to women at average risk of ovarian cancer.

Currently no reliable biomarker predicting the timing of menopause is available. This study used AMH as a surrogate marker of ovarian function. AMH was selected over FSH and estrogen due to its low intra‐ and inter‐cycle variability, permitting blood sampling independent of menstrual cycle phase.^29^ The level of AMH decreases with age until menopause; therefore, it has the potential to contribute to the prediction of menopause timing. In younger women, a faster decline in AMH is associated with a risk of lower age at menopause.^30^ The predictive effect, though, seems to be less strong with increasing age of the woman, and the predictive capacity of AMH on top of age alone decreases in women over 45 years of age,^31^, ^32^ This has to be considered the mean age of participants in our study was 45 years. Despite the low predictive value for AMH on individual timing of menopause, it can still be used to study the age of menopause in population studies.^33^

As surrogate measures of ovarian function have inherent limitations, it underscores the need for future studies to evaluate the actual impact of OS on age at menopause, menopausal symptoms, and subsequent hormone therapy use. We greatly anticipate the results of the Swedish HOPPSA trial^20^ and MISSION‐O (ClinicalTrials NCT07423143) examining menopause symptoms and FSH levels following OS.

This study's strengths include its prospective, randomized design and 12‐month follow‐up, making it the only trial to report ovarian effects over 1 year. However, randomization was designed for the HOPPSA trial, not for this subpopulation. Selection bias was avoided by asking for participation before randomization in HOPPSA. Data were retrieved from high‐quality registers with high coverage, though the registers have limitations, including missing data, lack of desirable variables, and the designs of the existing variables were not always optimal. To some extent, we could retrieve missing data or correct unreasonable values from medical records. AMH levels were measured using an immunoassay system characterized by low interassay coefficients of variation, indicating that the effect of analyzing samples at two different time points is likely negligible.

A limitation was the lack of information on when and if participants discontinued hormonal contraceptives, which could potentially influence the results as these drugs are known to have a reversible suppressive effect on AMH levels.^34^ Furthermore, a significant number of participants either did not respond to or did not receive the question regarding any estrogen treatment for menopause symptoms prior to surgery or at the 1‐year follow‐up, rendering this variable unreliable. Current smoking appears to suppress AMH levels by accelerating follicular depletion. This suppressive effect is dose‐dependent and the AMH decline rate is highest for current smokers, followed by former smokers and non‐smokers.^35^ The proportion of current smokers was relatively high in both study groups (20.0% in the OS group and 13.9% in the hysterectomy‐only group). Women are often encouraged to discontinue smoking prior to surgery because of its well‐established negative effects on postoperative healing. However, the lack of data on smoking behavior during the follow‐up period represents a limitation. Furthermore, obesity may negatively impact AMH production. Leptin is thought to play a role though the mechanisms are not fully understood. As adiposity increases, AMH production per antral follicle decreases.^36^ There was no preoperative difference in BMI observed in our study, though a loss or gain of weight during follow‐up time is unknown but is considered to have a minor impact on the results. Blinding of participants was not an issue in this substudy, as the endpoint was a hormonal analysis. The laboratory personnel were blinded to group allocation. We lack information on the number of women asked or who declined to participate, which limits generalizability. However, generalizability is likely to be high, especially in settings such as the Swedish society. In settings where other surgical methods are used, the generalizability may be lower since any effect on ovarian function could be dependent on the operative route or instruments used. Furthermore, there was a relatively large loss to follow‐up despite repeated reminders. However, no differences in baseline variables between participants lost to follow‐up and those who completed blood sampling were observed, making selective loss to follow‐up unlikely.

## 5 CONCLUSIONS

Noninferiority was not demonstrated for OS at hysterectomy compared with hysterectomy‐only regarding ovarian function assessed by AMH. Absolute reduction in AMH levels appeared to be similar in both groups, with an unexpectedly large variability in AMH reduction suggesting a more pronounced decline in some individuals.

## AUTHOR CONTRIBUTIONS

Annika Strandell, principal investigator (PI) of HOPPSA and Annika Idahl (co‐PI) conceived this sub‐study. Annika Strandell and Annika Idahl planned the design and wrote and published the study protocol together with Anna Darelius and Karin Sundfeldt. Anna Darelius recruited patients and managed follow‐up communications and wrote the statistical analysis plan based on discussions with the PIs and the HOPPSA‐responsible statistician. Anna Darelius had the primary contact with the statistician who conducted the final analyses. Anna Darelius wrote the first draft of the manuscript, which was critically reviewed and revised for intellectual content by all co‐authors. All authors have read and approved the final version of the manuscript.

## FUNDING INFORMATION

This research was funded by the Swedish Cancer Society (CAN 21 1408 PJ and CAN 21‐848 PJ), the Swedish state under the ALF agreement (ALFGBG‐971191 and ALFGBG‐965130), and the Jane and Dan Olsson Foundations (2016‐49). The funding sources were not involved in the collection, analysis, and interpretation of data, in the writing of the report, or in the decision to submit this article for publication.

## CONFLICT OF INTEREST STATEMENT

The authors have no conflicts of interest to disclose.

## ETHICS STATEMENT

The trial was approved by the Regional Ethical Review Board of Gothenburg, Sweden, 9 September 2016 (No. 501‐16 with additions T733‐17 and T407‐17). The trial was registered at clinicaltrials.gov (NCT03045965) on 8 February 2017. The date of initial participant enrollment was 16 August 2018 and informed consent was obtained from all research participants.

## Supporting information


**Table S1.** Anti‐Müllerian hormone (AMH) levels and changes in the per‐protocol population.
**Table S2.** Anti‐Müllerian hormone (AMH) levels and changes in the intention‐to‐treat population (multiple imputation of missing second samples).
**Table S3.** Anti‐Müllerian hormone (AMH) levels and changes in the as‐treated population (multiple imputation of missing second samples).
**Table S4.** Anti‐Müllerian hormone (AMH) levels and changes in women aged ≥50 years.
**Table S5.** Anti‐Müllerian hormone (AMH) levels and changes in women aged 45–49 years.
**Table S6.** Anti‐Müllerian hormone (AMH) levels and changes in women aged <45 years.

## Data Availability

The data that support the findings of this study are available on request from the corresponding author. The data are not publicly available due to privacy or ethical restrictions.
