# Supplementary material for: Effect of opportunistic salpingectomy at hysterectomy on anti‐Müllerian hormone: A substudy of a randomized trial
Source: Acta Obstet Gynecol Scand. 2026 May 19;105(8):1480–9. doi: 10.1111/aogs.70247 (PMC13356485; doi:10.1111/aogs.70247)
Supplement: Supplementary file 1 — Table S1. Anti‐Müllerian hormone (AMH) levels and changes in the per‐protocol population. Table S2. Anti‐Müllerian hormone (AMH) levels and changes in the intention‐to‐treat population (multiple imputation of missing second samples). Table S3. Anti‐Müllerian hormone (AMH) levels and changes in the as‐treated population (multiple imputation of missing second samples). Table S4. Anti‐Müllerian hormone (AMH) levels and changes in women aged ≥50 years. Table S5. Anti‐Müllerian hormone (AMH) levels and changes in women aged 45–49 years. Table S6. Anti‐Müllerian hormone (AMH) levels and changes in women aged <45 years. [file AOGS-105-1480-s001.docx]

**Table S1.** Anti-Müllerian hormone (AMH) levels and changes in the per-protocol population.

|  | Intervention | Control |
| --- | --- | --- |
| **A.** **Multiple imputation of missing second samples** | n=37 | n=37 |
| AMH-level pre-op, mean, µg/L (SD) | 1.01 (1.41) | 0.88 (2.00) |
| AMH-level 1-year post-op, mean(adj), µg/L (SD) | 0.70 (1.26) | 0.67 (1.62) |
| Absolute change in AMH, mean(adj), µg/L (SD) | -0.25 (0.62) | -0.28 (0.61) |
| Relative change in AMH, % (SD) | -14.3 (47.0) | -9.75 (72.0) |
| **B.** **Complete case analysis** | n=30 | n=32 |
| AMH-level pre-op, mean, µg/L (SD) | 1.01 (1.46) | 0.87 (2.13) |
| AMH-level 1-year post-op, mean, µg/L (SD) | 0.74 (1.26) | 0.62 (1.62) |
| Absolute change in AMH, mean(adj), µg/L (SD) | -0.25 (0.62) | -0.27 (0.61) |
| Median (Q1; Q3) | -0.16 (-0.30; -0.03) | -0.01 (-0.3; 0.01) |
| (Min; Max) | (-2.66; 0.60) | (-3.10; 0.20) |
| Relative change in AMH, % (SD) | -39.1 (47.0) | -7.18 (72.0) |
| Median (Q1; Q3) | -47.8 (-83.3; -8.0) | -5.86 (-55.83; 3.33) |
| (Min; Max) | (-93.5; 89.7) | (-92; 200) |

Adjustment variables: Baseline AMH, age groups, intended operative route and days from surgery to the second sampling of AMH.

**Table S2.** Anti-Müllerian hormone (AMH) levels and changes in the intention-to-treat population (multiple imputation of missing second samples).

|  | Intervention  n=46 | Control  n=43 |
| --- | --- | --- |
| AMH-level pre-op, mean, µg/L (SD) | 0.93 (1.30) | 0.82 (1.86) |
| AMH-level 1-year post-op, mean(adj), µg/L (SD) | 0.58 (1.15) | 0.61 (1.51) |
| Absolute change in AMH, mean(adj), µg/L (SD) | -0.28 (0.59) | -0.26 (0.57) |
| Relative change in AMH, % (SD) | -44.4 (45.0) | -9.90 (70.5) |

Adjustment variables: Baseline AMH, age groups, intended operative route and days from surgery to the second sampling of AMH.

**Table S3.** Anti-Müllerian hormone (AMH) levels and changes in the as-treated population (multiple imputation of missing second samples).

|  | Intervention  n=39 | Control  n=41 |
| --- | --- | --- |
| AMH-level pre-op, mean, µg/L (SD) | 1.00 (1.37) | 0.84 (1.90) |
| AMH-level 1-year post-op, mean(adj), µg/L (SD) | 0.66 (1.25) | 0.62 (1.53) |
| Absolute change in AMH, mean(adj), µg/L (SD) | -0.25 (0.61) | -0.29 (0.57) |
| Relative change in AMH, % (SD) | -39.7 (46.5) | -15.0 (71.4) |

Adjustment variables: Baseline AMH, age groups, intended operative route and days from surgery to the second sampling of AMH.

**Tables S4-6.** Anti-Müllerian hormone (AMH) levels and changes in age strata of the per-protocol population.

**Table S4.** Anti-Müllerian hormone (AMH) levels and changes in women ≥50 years.

|  | Intervention | Control |
| --- | --- | --- |
| **A.** **Multiple imputation of missing second samples** | n=9 | n=10 |
| AMH-level pre-op, mean, µg/L (SD) | 0.15 (0.20) | 0.07 (0.09) |
| AMH-level 1-year post-op, mean(adj), µg/L (SD) | 0.05 (0.12) | 0.04 (0.01) |
| Absolute change in AMH, mean(adj), µg/L (SD) | -0.06 (0.11) | -0.06 (0.09) |
| Relative change in AMH, % (SD) | 67.8 (35.4) | -7.98 (94.0) |
| **B.** **Complete case analysis** | n=6 | n=9 |
| AMH-level pre-op, mean, µg/L (SD) | 0.21 (0.23) | 0.07 (0.09) |
| AMH-level 1-year post-op, mean, µg/L (SD) | 0.07 (0.12) | 0.03 (0.01) |
| Absolute change in AMH, mean(adj), µg/L (SD) | -0.09 (0.11) | -0.08 (0.09) |
| Median (Q1:Q3) | -0.12 (-0.22; 0.07) | 0 (-0.01; 0.01) |
| (Min; Max) | (-0.32; 0) | (-0.23; 0.02) |
| Relative change in AMH, % (SD) | -45.9 (35.4) | 0.73 (94.0) |
| Median (Q1; Q3)  (Min; Max) | -81 (-87.5; -50)  (-92.9; 0) | 0 (-50.0; 66.7)  (-92; 200) |

Adjustment variables: Baseline AMH, age groups, intended operative route and days from surgery to the second sampling of AMH.

**Table S5.** Anti-Müllerian hormone (AMH) levels and changes in women 45-49 years.

|  | Intervention | Control |
| --- | --- | --- |
| **A.** **Multiple imputation of missing second samples** | n=12 | n=14 |
| AMH-level pre-op, mean, µg/L (SD) | 0.53 (0.98) | 0.34 (0.55) |
| AMH-level 1-year post-op, mean(adj), µg/L (SD) | 0.11 (0.27) | 0.26 (0.15) |
| Absolute change in AMH, mean(adj), µg/L (SD) | -0.32 (0.76) | -0.17 (0.19) |
| Relative change in AMH, % (SD) | -67.6 (23.9) | -0.08 (72.6) |
| **B.** **Complete case analysis** | n=11 | n=12 |
| AMH-level pre-op, mean, µg/L (SD) | 0.56 (1.02) | 0.22 (0.24) |
| AMH-level 1-year post-op, mean, µg/L (SD) | 0.14 (0.27) | 0.16 (0.15) |
| Absolute change in AMH, mean(adj), µg/L (SD) | -0.29 (0.76) | -0.17 (0.19) |
| Median (Q1; Q3)  (Min; Max) | -0.21 (-0.35; -0.05)  (-2.66; -0.02) | 0 (-0.07; 0.02)  (-0.59; 0.20) |
| Relative change in AMH, % (SD) | -64.7 (23.9) | 4.53 (72.6) |
| Median (Q1; Q3)  (Min; Max) | -73.9 (-84; -57.1)  (-93.5; -14.3) | 0 (-16.8; 11.9)  (-90.48; 200) |

Adjustment variables: Baseline AMH, age groups, intended operative route and days from surgery to the second sampling of AMH.

|  | Intervention | Control |
| --- | --- | --- |
| **A.** **Multiple imputation of missing second samples** | n=16 | n=13 |
| AMH-level pre-op, mean, µg/L (SD) | 1.86 (1.62) | 2.08 (3.04) |
| AMH-level 1-year post-op, mean(adj), µg/L (SD) | 1.70 (1.59) | 1.36 (2.54) |
| Absolute change in AMH, mean(adj), µg/L (SD) | -0.27 (0.65) | -0,60 (0.91) |
| Relative changeadj in AMH, % (SD) | -9.29 (43.5) | -35.3 (38.4) |
| AMH-level pre-op, mean, µg/L (SD) | 1.86 (1.62) | 2.08 (3.04) |
| **B.** **Complete case analysis** | n=13 | n=11 |
| AMH-level pre-op, mean, µg/L (SD) | 1.76 (1.79) | 2.24 (3.29) |
| AMH-level 1-year post-op, mean, µg/L (SD) | 1.55 (1.59) | 1.60 (2.54) |
| Absolute change in AMH, mean(adj), µg/L (SD) | -0.22 (0.65) | -0.63 (0.91) |
| Median (Q1; Q3) | -0.10 (-0.20; 0.13) | -0.43 (-0.80; -0.03) |
| (Min; Max) | (-1.60; 0.60) | (-3.10; 0.18) |
| Relative changeadj in AMH, % (SD) | -6.23 (43.5) | -35.9 (38.4) |
| Median (Q1; Q3) | -8.0 (-42.86; 17.81) | -40.0 (-77.8; -2.0) |
| (Min; Max) | (-49.35; 89.74) | (-82; 38.3) |

**Table S6.** Anti-Müllerian hormone (AMH) levels and changes in women <45 years.

Adjustment variables: Baseline AMH, age groups, intended operative route and days from surgery to the second sampling of AMH.
